# Blood pressure variability associates with mortality risk of patients with ANCA-associated vasculitis

**DOI:** 10.1093/ckj/sfag356

**Published:** 2026-10-07

**Authors:** Elisabetta Bussalino, Elisa Russo, Lucia Maccio, Marta Donati, Federica Mescia, Marta Calatroni, Martina Tedesco, Gabriella Moroni, Federico Alberici, Francesca Viazzi, Pasquale Esposito

**Affiliations:** Nephrology, dialysis and Transplantation Unit, IRCCS Azienda Ospedaliera Metropolitana, Genova, Italy; Nephrology, dialysis and Transplantation Unit, IRCCS Azienda Ospedaliera Metropolitana, Genova, Italy; Department of Internal Medicine and Medical Specialties (DIMI), University of Genova, Genova, Italy; Nephrology, dialysis and Transplantation Unit, IRCCS Azienda Ospedaliera Metropolitana, Genova, Italy; Department of Internal Medicine and Medical Specialties (DIMI), University of Genova, Genova, Italy; Department of Internal Medicine and Medical Specialties (DIMI), University of Genova, Genova, Italy; Nephrology Unit, Università degli Studi di Brescia–ASST Spedali Civili di Brescia, Brescia, Italy; Nephrology and Dialysis Division, IRCCS Humanitas Research Hospital, Milano, Italy; Department of Biomedical Sciences, Humanitas University, Pieve Emanuele, Milano, Italy; Nephrology Unit, Università degli Studi di Brescia–ASST Spedali Civili di Brescia, Brescia, Italy; Nephrology and Dialysis Division, IRCCS Humanitas Research Hospital, Milano, Italy; Department of Biomedical Sciences, Humanitas University, Pieve Emanuele, Milano, Italy; Nephrology Unit, Università degli Studi di Brescia–ASST Spedali Civili di Brescia, Brescia, Italy; Nephrology, dialysis and Transplantation Unit, IRCCS Azienda Ospedaliera Metropolitana, Genova, Italy; Department of Internal Medicine and Medical Specialties (DIMI), University of Genova, Genova, Italy; Nephrology, dialysis and Transplantation Unit, IRCCS Azienda Ospedaliera Metropolitana, Genova, Italy; Department of Internal Medicine and Medical Specialties (DIMI), University of Genova, Genova, Italy

**Keywords:** antineutrophil cytoplasmic antibody-associated vasculitis, blood pressure control, endothelial dysfunction, inflammation, mortality risk, systolic blood pressure variability, treatment

## Abstract

**Objectives:**

Antineutrophil cytoplasmic antibody‐associated vasculitis (AAV) is associated with increased cardiovascular risk, related to persistent inflammation and endothelial dysfunction. Long‐term systolic blood pressure variability (SBPV), reflecting vascular damage, may predict outcomes better than mean blood pressure (BP). We investigated the impact of BP control and visit-to-visit BP variability (VVV), on all-cause mortality in AAV patients during maintenance therapy.

**Methods:**

We retrospectively studied 95 AAV patients followed at three Italian nephrology centers. SBPV was defined as the standard deviation of ≥4 systolic BP measurements within 12 months from the first SBPV assessment. Patients were stratified by SBPV tertiles. The primary outcome was all-cause mortality.

**Results:**

Over a median follow-up of 78 months, 14 patients (14.7%) died. Deceased patients were older and had higher pulse pressure (PP) and VVV. Hypertension prevalence increased across SBPV tertiles (35%, 62%, 69%; *P* = .02). Mortality rose from 6% in the lowest to 34.5% in the highest SBPV tertile (Fisher’s exact, *P* = .001). Time-averaged systolic BP was not associated with mortality. Conversely, each 5-mmHg increase in SBPV was associated with a two-fold higher risk of death [hazard ratio (HR) 2.03, 95% confidence interval (CI) 1.29–3.20; *P* = .002]. SBPV remained significantly associated with outcome after full multi-variable adjustment, including age, sex, myeloperoxidase positivity, estimated glomerular filtration rate, PP, and proteinuria (adjusted HR 8.06 per 1 mmHg, 95% CI 1.42–45.54; *P* = .02).

**Conclusion:**

In AAV patients, increased VVV was associated with all-cause mortality outperforming mean systolic BP. Further research is needed to confirm the impact of variable blood pressure on patient outcomes in AAV.

## Graphical Abstract

**Graphical Abstract:**
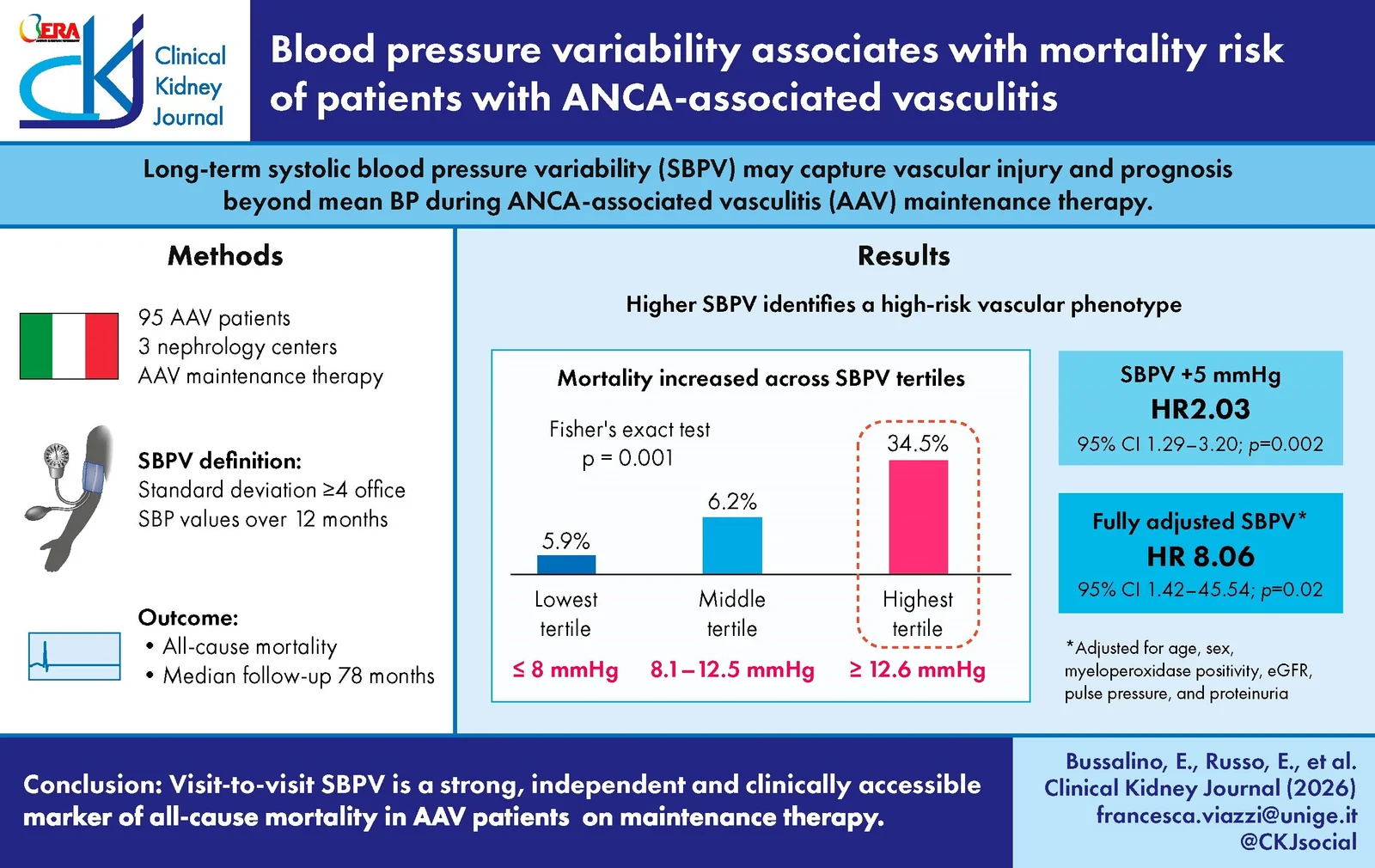

KEY LEARNING POINTS **What was known:**

- ANCA-associated vasculitis is characterized by excess long-term mortality, largely attributable to cardiovascular disease related to persistent inflammation, endothelial dysfunction, and renal impairment. Blood pressure is a major modifiable risk factor, but mean systolic values alone incompletely reflect vascular damage and long-term cardiovascular risk.

**This study adds:**

- In AAV patients during maintenance therapy, visit-to-visit systolic blood pressure variability (SBPV) independently predicts all-cause mortality, with a dose–response relationship. SBPV provides prognostic information beyond age, renal function, pulse pressure, autoantibody profile, and mean blood pressure levels.

**Potential impact:**

- Blood pressure variability represents a clinically accessible surrogate of vascular injury in AAV. Routine monitoring of SBPV may enable earlier identification of high-risk patients and promote targeted strategies to stabilize blood pressure, optimize antihypertensive therapy, and potentially reduce long-term cardiovascular and mortality risk.

## INTRODUCTION

Antineutrophil cytoplasmic antibody-associated vasculitis (AAV) comprises a group of severe systemic disorders characterized by small-vessel necrotizing vasculitis and shared pathogenic mechanisms [1]. Clinical presentation is often systemic, with frequent multiorgan involvement, mostly affecting the kidneys, respiratory tract, gastrointestinal system, and skin [2].

AAV typically follows an aggressive clinical course, with renal involvement manifesting as rapidly progressive glomerulonephritis [3], historically associated with extremely poor outcomes and 1-year mortality rates approaching 90% [4]. However, the advent of novel therapeutic approaches, primarily based on an improved understanding of underlying pathophysiological mechanisms, such as the targeting of inflammatory pathways, B-cell function, and, more recently, complement activation, has significantly transformed disease management [5]. The introduction of effective immunosuppressive strategies has led to a dramatic improvement in survival, with current 1-year survival rates close to 90% [6].

These developments have led to a paradigm shift in the treatment and management of AAV [7]. While research continues to focus on optimizing induction therapy, particularly aimed at minimizing treatment-related risks, increasing attention has been directed toward the maintenance phase of treatment [8, 9], whose primary objective is the prevention of disease recurrence, an occurrence that may impact patients’ prognosis [10]. Despite these therapeutic gains, AAV remains a life-threatening condition with long-term mortality substantially exceeding that of the general population [11]. While infections and active vasculitis dominate early mortality, cardiovascular disease (CVD) emerges as the leading cause of death beyond the first year [12]. AAV patients experience a nearly four-fold higher cardiovascular (CV) mortality compared with the general population, reflecting a synergistic burden of chronic inflammation, endothelial dysfunction, accelerated atherosclerosis, and frequent renal involvement [13–16]. Thus, although immunosuppression remains the primary treatment to control inflammation and prevent organ damage, careful management of CV risk factors constitutes a critical component of long-term care. Nevertheless, despite growing awareness of these issues and their prognostic implications, research focused on CV risk factors in AAV remains limited.

In this context, long-term visit-to-visit BP variability (VVV) has emerged as a clinically relevant marker that integrates hemodynamic instability and vascular health. Beyond reflecting blood pressure (BP) control, elevated VVV is increasingly recognized as a surrogate of vascular aging and arterial stiffness, representing cumulative vascular injury rather than transient BP elevation [17]. Previous studies have demonstrated that VVV is a strong predictor of CV outcomes and renal disease progression in various clinical contexts, including diabetes, Immunoglobulin A (IgA) nephropathy, non-dialysis chronic kidney disease (CKD) [18–20], and transplant recipients [21]. Considering the current lack of specific data in AAV populations, the present study aimed to investigate the impact of VVV on mortality risk in a cohort of AAV patients undergoing maintenance therapy.

## MATERIALS AND METHODS

### Study sample

We conducted a retrospective analysis of patients with AAV receiving maintenance therapy between 1 January 2001 and 1 January 2020, followed at three outpatient nephrology clinics (IRCCS San Martino Polyclinic Hospital, Brescia Civil Hospital, and IRCCS Humanitas Hospital). Inclusion required at least four visits with office BP measurements and available clinical and laboratory data within the 12 months used for systolic blood pressure variability (SBPV) assessment.

### Study design

Data regarding demographics, immunological data, coexisting comorbidities, and information on medications were obtained for all enrolled patients at the vasculitis diagnosis and at the first assessment of SBPV (i.e. after completion of the AAV induction phase), and thereafter updated at every follow-up visit by self-reports and review of medical records (Fig. 1).

**Figure 1:**
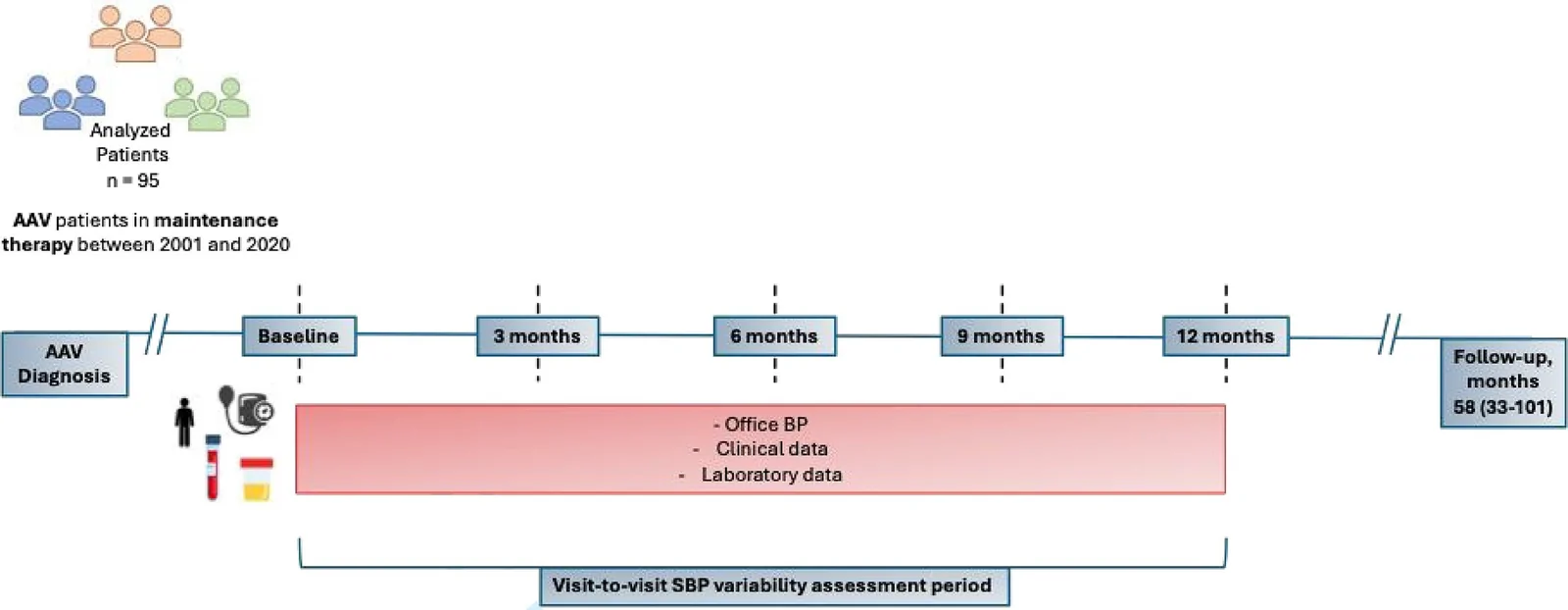
Study design.

All patients were diagnosed with AAV, including microscopic polyangiitis, granulomatosis with polyangiitis, or renal-limited vasculitis, according to the American College of Rheumatology classification criteria and the Chapel Hill Consensus Conference definitions [22]. Patients were included if they had either histologically proven vasculitis with renal involvement and/or serological evidence of Anti-Neutrophil Cytoplasmic Antibodies (ANCA) positivity, specifically myeloperoxidase (MPO)-ANCA or proteinase 3-ANCA. Renal involvement was defined by rapidly progressive glomerulonephritis, characterized by a rapid decline in kidney function and/or the presence of active urinary sediment with hematuria and glomerular proteinuria.

Kidney biopsy, when clinically feasible, was performed at diagnosis or within 1 month of disease onset or renal flare, and histopathological findings were classified according to the Berden histopathological classification.

Laboratory measurements included hemoglobin, uric acid, creatinine, glucose, total cholesterol, C-reactive protein, and 24-h urinary protein excretion. Renal function, as expressed by estimated glomerular filtration rate (eGFR), was calculated according to the Chronic Kidney Disease Epidemiology Collaboration (CKD-EPI) formula [23]. Diabetes was defined as a self-reported diagnosis or use of hypoglycemic drugs.

Prior CVD was identified by reviewing previous inpatient hospital records as a history of myocardial infarction, previous coronary revascularization, or stroke. Clinical data were collected at the time of AAV diagnosis, as reported in Table 1, and subsequently at the initiation of maintenance therapy, corresponding to the first assessment of SBPV, as reported in Table 2. All procedures were in accordance with the ethical standards of the institutional and/or national research committee and with the 1964 Helsinki Declaration and its later amendments. Although detailed data on glucocorticoid dosage during induction therapy were not consistently available, patients were treated according to contemporary international guideline recommendations for AAV (including EULAR guidelines), ensuring a broadly homogeneous therapeutic approach across all participating centers. The study was carried out under the ethics of the “Coorte MaRe” project, approved by the Ethics Committee of ASST Spedali Civili di Brescia (ethics number 4945). Written informed consent was obtained from all the participants.

**Table 1:** Main characteristic of the study population at AAV diagnosis stratified by SBPV tertiles.

Variable	All	Low (<8 mmHg)	Middle (8.1–12.5 mmHg)	High (>12.6 mmHg)	*P*
*N*	95	34	32	29	
Age, years	68.44 ± 14.79	66.35 ± 17.09	67.15 ± 12.18	72.31 ± 14.26	.42
Sex, female, *n* (%)	47 (49)	17 (50)	19 (59)	11 (38)	.26
Age at onset, years	60.43 ± 16.36	57.35 ± 17.89	60.03 ± 14.93	64.48 ± 15.56	.52
Pulmonary involvement, *n* (%)	41 (43)	18 (52)	14 (43)	9 (31)	.21
MPO/PR3/negative, *n* (%)	57 (60)/31 (33)/7 (7)	17 (50)/15 (44)/2 (6)	23 (71)/6 (18)/3 (1)	17 (59)/10 (34)/2 (7)	.27
RRT at onset	12 (13)	5 (15)	5 (16)	2 (7)	.54
With hypertension, *n* (%)	**52 (55)**	**12 (35)**	**20 (62)**	**20 (69)**	**.02**
With diabetes, *n* (%)	9 (9)	3 (9)	5 (16)	1 (3)	.25
With previous cardiovascular disease, *n* (%)	3 (3)	2 (6)	1 (3)	0 (0)	.77
Creatinine (mg/dl)	3.84 ± 2.51	4.11 ± 2.76	3.85 ± 2.79	3.52 ± 1.84	.65
eGFR, ml/min/1.73 m^2^ (IQR)	16 (22)	15 (18)	19 (18)	15 (22)	.61
Proteinuria, g/24 h, (IQR)	1 (1.30)	0.82 (1.16)	1.00 (1.15)	1.06 (1.1)	.72
CPR, mg/l (IQR)	19.79 (39.7)	20.89 (37.3)	16.60 (45)	20.15 (45)	.96
C3, mg/dl	81.80 ± 27.11	90.00 ± 10.53	83.47 ± 32.06	77.33 ± 16.53	.21
C4, mg/dl	30.61 ± 10.74	30.45 ± 6.46	27.93 ± 12.21	34.61 ± 10.98	.73
Induction therapy					
IV CYC	37 (39)	15 (44)	12 (38)	10 (34)	.72
OS CYC	32 (34)	12 (35)	6 (19)	14 (48)	.06
RTX	**41 (43)**	**14 (41)**	**19 (59)**	**8 (28)**	**.04**
PEX	20 (21)	9 (26)	5 (16)	6 (21)	.60
Maintenance therapy					
On azathioprine, *n* (%)	38 (40)	14 (41)	14 (44)	10 (34)	.78
On MMF, *n* (%)	28 (29)	9 (26)	9 (28)	10 (34)	.81
On steroid, *n* (%)	92 (96)	33 (97)	30 (94)	29 (100)	.64

Bold values indicate statistically significant results (P < 0.05). Abbreviations: CRP, C-reactive protein; IV CYC, intravenous cyclophosphamide; eGFR, estimated glomerular filtration rate (ml/min/1.73 m²); IQR, interquartile range; MMF, mycophenolate mofetil; MPO, myeloperoxidase antibodies; OS CYC, oral cyclophosphamide; PEX, plasma exchange; PR3, proteinase 3 antibodies; RRT, renal replacement therapy; RTX, rituximab.

**Table 2:** Main characteristic of the study population at the first-time SBPV assessment stratified by SBPV tertiles.

Variable	All	Low (<8 mmHg)	Middle (8.1–12.5 mmHg)	High (>12.6 mmHg)	*P*
*N*	95	34	32	29	
Time from AAV diagnosis to first-time SBPV assessment visit, months (IQR)	11 (9–13)	10 (9–13)	11 (9–15)	10 (8–13)	.67
Creatinine (mg/dl)	1.49 ± 1.19	1.38 ± 1.01	1.5 ± 1.21	1.62 ± 1.37	.50
eGFR, ml/min/1.73m^2^ (IQR)	39.05 ± 21.95	39.81 ± 22.91	38.13 ± 18.83	39.17 ± 24.47	.50
Proteinuria, g/24 h, (IQR)	1.00 (1.30)	0.82 (1.2)	1.00 (1.13)	1.06 (1.0)	.13
CPR, mg/l	19.54 ± 21.80	16.45 ± 15.32	17.42 ± 18.39	24.54 ± 30.08	.74
Hemoglobin, g/dl	11.84 ± 1.30	11.90 ± 1.89	11.90 ± 1.51	11.71 ± 1.30	.10
Uric acid, mg/dl	5.68 ± 1.76	5.23 ± 1.53	5.5 ± 1.68	6.35 ± 1.98	.46
Glucose, mg/dl	93.26 ± 23.13	86.25 ± 12.80	91.85 ± 18.97	102.75 ± 34.94	.94
Total cholesterol, mg/dl	222.7 ± 44.45	226.33 ± 54.23	223.11 ± 33.61	217.58 ± 48.66	.50
SBP, mmHg	140.05 ± 16.99	135.37 ± 14.59	137.33 ± 15.01	147.55 ± 19.01	.17
DBP, mmHg	82.60 ± 10.08	84.51 ± 8.17	80.83 ± 10.42	82.51 ± 11.38	.31
PP, mmHg	57.05 ± 15.52	51.41 ± 11.60	55.81 ± 12.94	65.03 ± 18.93	.11
TASBP, mmHg	**138.24 ± 12.99**	**136.07 ± 13.16**	**135.84 ± 11.67**	**143.45 ± 13.09**	**.03**
TAPP, mmHg	55.85 ± 12.48	52.76 ± 11.70	53.59 ± 9.74	61.96 ± 14.17	.19
Number of antihypertensive drugs, *n* (IQR)	1 (1)	1 (2.5)	1 (2)	1 (1)	.62
On beta-blockers, *n* (%)	22 (23)	10 (29)	9 (28)	3 (10)	.30
On RAAS blockers, *n* (%)	18 (19)	7 (21)	7 (22)	4 (14)	1.00
On statin, *n* (%)	16 (17)	4 (12)	10 (31)	2 (7)	.15

Bold values indicate statistically significant results (P < 0.05). Abbreviations: AAV, ANCA-associated vasculitis; CRP, C-reactive protein; DBP, diastolic blood pressure; eGFR, estimated glomerular filtration rate (ml/min/1.73 m²); IQR, interquartile range; SBP, systolic blood pressure; PP, pulse pressure; RAAS, renin–angiotensin system; TAPP, time-averaged pulse pressure; TASBP, time-averaged systolic blood pressure.

The primary outcome was all-cause mortality. All events were identified through a comprehensive review of patients’ medical records. The event-free survival was defined as the time from the last variability assessment visit to the first event or the censoring date (1 February 2024). The study follow-up lasted a median time of 58 months (range 33–101 months).

### Blood pressure and visit-to-visit systolic variability

At each visit, office systolic BP (SBP) and diastolic BP (DBP) were measured in each patient according to the ESC/ESH guidelines [24] by the means of a validated aneroid sphygmomanometer. BP was assessed after 5 min of rest, in a sitting position, three times at 2-min intervals. The average of the second and third readings was used to define SBP and DBP, excluding the first measurement. The average value was used to define SBP and DBP. Pulse pressure (PP) was defined as the difference between SBP and DBP [17]. Hypertension was defined as BP higher or equal to 140/90 mmHg or when patients were taking antihypertensive medications at disease onset.

During the maintenance phase of AAV, ∼1 year after diagnosis, visit-to-visit SBPV was calculated as the SD of at least four SBP measurements obtained at the first assessment visit and subsequently every 3 months over a 12-month assessment period (Fig. 1).

Time-averaged systolic BP (TASBP) and time-averaged PP (TAPP) were examined as the average of all available SBP and PP values during the assessment period, respectively. The patients were categorized into tertiles based on SBPV, with cut-offs values (8.1 and 12.5 mmHg) derived from the distribution of SBPV within the study population.

### Statistical analysis

Continuous variables are presented as means with SDs for normally distributed data and compared using *t*-test or one-way ANOVA. Skewed data were logarithmically transformed or expressed as median with interquartile range and compared with the Kruskal–Wallis test. The normal distribution of the variables was determined by the Kolmogorov–Smirnov test. Categorical variables are presented as the number of patients with a proportion and compared with Fisher’s exact test as appropriate. The median follow-up time was estimated using the reverse Kaplan–Meier method. Kaplan–Meier analysis with log-rank test was carried out to compare the event-free survival curves according to visit-to-visit SBP tertiles. Only the first events were considered.

The association between SBPV and the study end point was assessed using univariate and multi-variable Cox proportional hazards regression analyses, with SBPV modeled as a continuous variable in the primary analyses. Incremental multi-variable models were constructed as described below. Covariates were selected based on both statistical significance in the univariate analysis (*P* < .05) and established clinical relevance to all-cause mortality. An additional Cox regression comparing patients in the highest SBPV tertile with those in the combined lower two tertiles was performed as a supplementary analysis (Table S3).

Results are reported as hazard ratios (HRs) with 95% confidence intervals (CIs) and *P* values. The proportional hazards assumption was verified using Schoenfeld residuals. Model discrimination was assessed using Harrell’s *C* statistic. Statistical calculations were performed by STATA package, version 14.2 (StataCorp, College Station, TX).

## RESULTS

### Patient characteristics

A total of 95 patients were included in the analysis. The main clinical characteristics of the study population at the AAV diagnosis and at the time of the first assessment of SBPV are summarized in Tables 1 and 2, respectively, stratified by SBPV tertiles.

Overall, the mean age was 68 ± 14, 51% of patients were men, 55% had hypertension, and 9% diabetes. Nearly 43% of patients had pulmonary involvement, MPO-ANCA was the most frequent autoantibody reported (60%), and ∼13% of patients underwent hemodialysis at disease onset. Induction therapy consisted of rituximab or cyclophosphamide (administered either intravenously or orally). Notably, patients with higher SBPV less frequently received rituximab as induction therapy.

Maintenance therapy commonly included corticosteroids, azathioprine, rituximab, and mycophenolate. The median time from diagnosis to the first-time SBPV assessment visit was 11 months.

At this time point, mean office BP was 140 ± 16/82±10 mmHg. Compared with the evaluation at AAV diagnosis, kidney function had significantly improved (eGFR 39 ± 21 ml/min/1.73 m²), while proteinuria remained stable at ∼1 g/24 h (Table 2).

Of note, patients with the highest SBPV were more likely to be hypertensive at AAV diagnosis and showed significantly higher TASBP during follow-up. Conversely, no statistically significant differences were observed in other baseline comorbidities or in the glyco-metabolic profile at the start of the assessment period across SBPV tertiles (Tables 1 and 2).

### Follow-up and outcome

Median follow-up, estimated using the reverse Kaplan–Meier method, was 78 months. During follow-up, 14 patients (14.7%) died with an incidence rate of 30.5 events per 1000 patient-years. Patients who experienced the end point were older at the time of AAV diagnosis compared with survivors (Table S1).

At the time of the first SBPV assessment (i.e. after completion of the AAV induction phase), patients who subsequently died had significantly higher PP and greater VVV (SBPV 13.98 ± 4.6 vs 10.03 ± 4.56, *P* = .003). In contrast, there was no significant difference in TASBP (137.9 ± 12.9 vs 140.5 ± 14.3, *P* = .49) and TAPP (55.4 ± 11.6 vs 60.9 ± 16.2, *P* = .12) between the two groups (Table 3).

**Table 3:** Patient characteristics at first-time SBPV by mortality status.

Variable	All	Deceased	Survivors	*P*
*N*	95	14	81	
Age, years	68.77 ± 14.51	76.78 ± 11.57	67.37 ± 14.57	.02
Creatinine (mg/dl)	1.94 ± 1.17	2.11 ± 1.15	1.91 ± 1.15	.55
eGFR, ml/min/1.73 m^2^ (IQR)	39 (21–55)	34 (19–49)	39 (21–56)	.31
Proteinuria, g/24 h, (IQR)	1.0 (0.5–1.8)	1.10 (0.4–1.51)	0.91 (0.50–1.82)	.74
CPR, mg/l	19.79 (3.3–43)	21.1 (4.58–89.5)	18.00 (3.3–38.9)	.78
Haemoglobin, g/dl	12.28 ± 1.57	11.74 ± 1.44	12.38 ± 1.58	.16
Uric acid, mg/dl	6.23 ± 1.75	6.06 ± 0.77	6.25 ± 1.84	.82
Glucose, mg/dl	93.55 ± 23.48	114.25 ± 48.5	90.24 ± 16.33	.06
Total cholesterol, mg/dl	223.25 ± 44.82	192.66 ± 30.36	225.48 ± 45.14	.22
SBP, mmHg	139.82 ± 16.5	144.53 ± 23.99	139 ± 15.46	.27
DBP, mmHg	82.45 ± 10.05	78.30 ± 5.39	83.18 ± 10.52	.10
PP, mmHg	57.36 ± 15.95	66.23 ± 27.23	55.81 ± 12.71	.03
TAPP, mmHg	56.17 ± 12.44	60.9 ± 16.2	55.4 ± 11.6	.12
TASBP, mmHg	138.24 ± 12.99	140.5 ± 14.3	137.9 ± 12.9	.49
Number of antihypertensive drugs, *n* (IQR)	1 (1–2)	1(0–3)	1 (1–2)	.87
On beta-blockers, *n* (%)	21 (22)	1 (7)	20 (24)	.41
On RAAS blockers, *n* (%)	17 (18)	2 (14)	15 (18)	1.00
On statin, *n* (%)	15 (16)	1 (7)	14 (17)	1.00

Abbreviations: CRP, C-reactive protein; DBP, diastolic blood pressure; eGFR, estimated glomerular filtration rate (ml/min/1.73 m²); IQR, interquartile range; PP, pulse pressure; RAAS, renin–angiotensin system; SBP, systolic blood pressure; TAPP, time-averaged pulse pressure; TASBP, time-averaged systolic blood pressure.

Kidney function trajectory was also evaluated according to SBPV categories. The monthly eGFR slope from the last visit included in the SBPV assessment period to the end of follow-up did not differ significantly between patients in the combined lower two SBPV tertiles and those in the highest SBPV tertile (−0.16 ± 3.91 vs 0.04 ± 0.47 ml/min/1.73 m²/month; *P* = .79). Similarly, the monthly eGFR slope during the SBPV assessment period, from the first to the last BP observation, was comparable between the two groups (−0.05 ± 3.66 vs 0.05 ± 0.20 ml/min/1.73 m²/month; *P* = .88).

In the univariate Cox regression analysis, older age at disease onset and higher proteinuria were significantly associated with all-cause mortality. Each 5 mmHg increase in baseline PP was associated with a 19% higher risk of death. Additionally, each 5 mmHg increase in SBPV was associated with a two-fold higher mortality risk (HR 2.03, 95% CI 1.29–3.20, *P* = .002), Kaplan–Meier analysis demonstrated significantly lower event-free survival in patients within the highest SBPV tertile compared with those in the combined lower two tertiles (Fig. 2; log-rank *P* = .0003).

**Figure 2:**
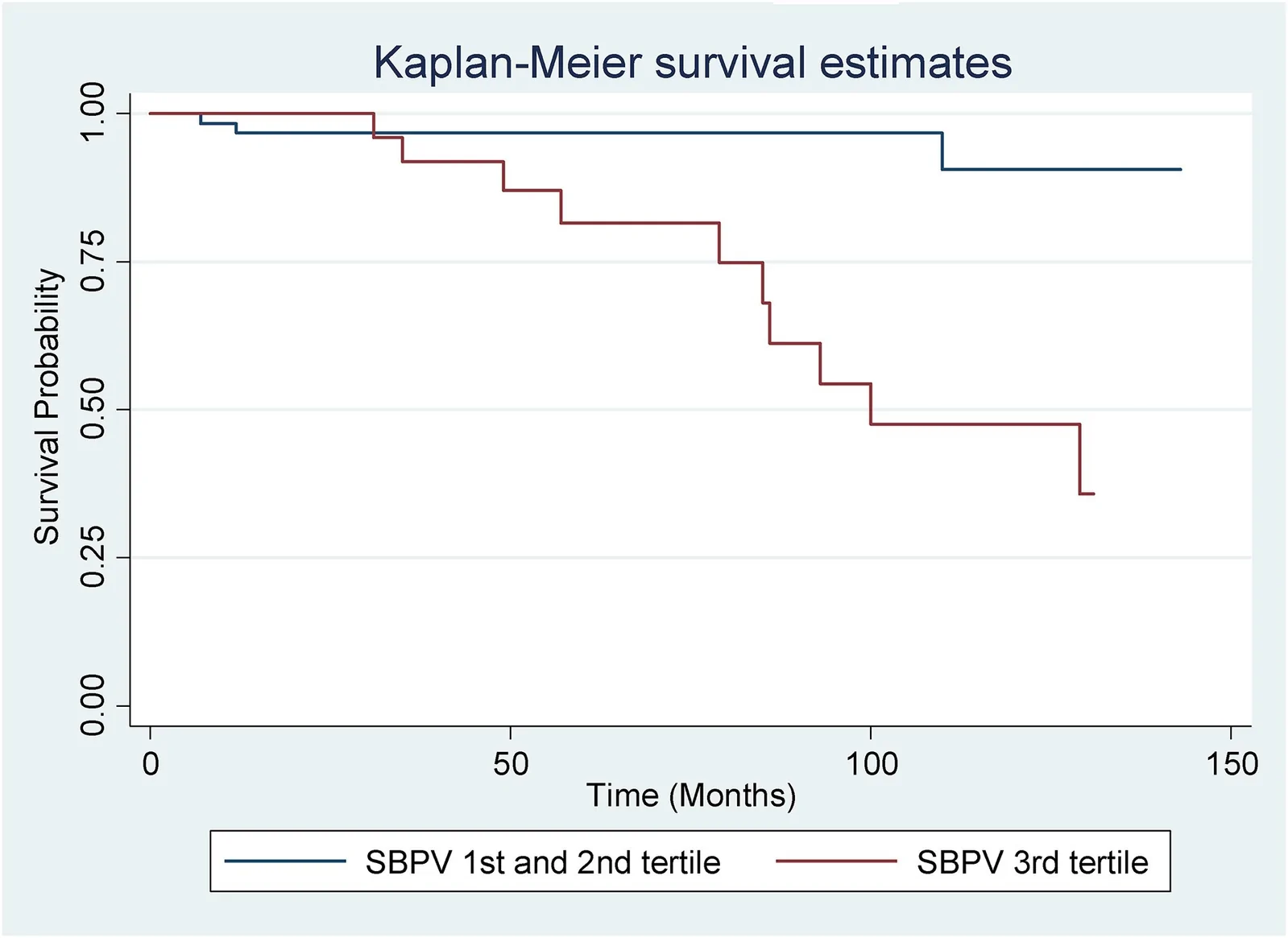
Kaplan–Meier curves for all-cause mortality according to SBPV tertiles. Patients were stratified into tertiles based on SBPV distribution: ≤12.5 mmHg (low and intermediate variabilities, first and second SBPV tertiles), and ≥12.6 mmHg (high variability, third SBPV tertile). Survival differed significantly between groups (log-rank *P* = .0003), with higher SBPV associated with increased all-cause mortality.

The association between SBPV and all-cause mortality remained significant after incremental multi-variable adjustment for PP (Model 1: Harrell’s *C*, 0.73, AIC 88.3), age at disease onset and gender (Model 2: Harrell’s *C*, 0.78, Akaike Information Criterion (AIC) 90.7), MPO positivity, eGFR, and 24-h proteinuria (Model 3) (Table 4). Model 3 showed good discriminatory performance (Harrell’s *C*, 0.82, AIC 94.0).

**Table 4:** Univariate and multivariate Cox regression analysis for all-cause mortality.

	Univariate		Model 1		Model 2		Model 3	
Variables	HR (CI 95%)	*P*	HR (CI 95%)	*P*	HR (CI 95%)	*P*	HR (CI 95%)	*P*
SBPV, mmHg	**8.19 (2.07–32.29)**	**.01**	**7.18 (1.74–29.53)**	**.01**	**7.71 (1.84–32.22)**	**.01**	**8.06 (1.42–45.54)**	**.02**
PP, mmHg	**1.19 (1.02–1.39)**	**.02**	1.02 (0.98–1.06)	.22	0.99 (0.95–1.06)	.99	1.01 (0.96–1.08)	.62
Female sex	1.25 (0.42–3.73)	.68	–	–	1.72 (0.52–5.58)	.37	2.49 (0.61–10.09)	.19
Age at onset, years	1.06 (0.01–1.10)	.01	–	–	1.03 (0.97–1.10)	.29	1.04 (0.96–1.13)	.28
eGFR, ml/min/1.73 m^2^	0.97(0.95–1.00)	.08	–	–	–	–	0.97 (0.91–1.02)	.29
Proteinuria, g/24 h,	**1.48(1.00–2.18)**	**.04**	–	–	–	–	1.26 (0.79–2.01)	.32
MPO positive	1.26 (0.39–4.14)	.56	–	–	–	–	1.91(0.56–34.90)	.71

Bold values indicate statistically significant results (P < 0.05). Abbreviations: CI, confidence intervals; eGFR, estimated glomerular filtration rate (ml/min/1.73 m²); HR, hazard ratio; MPO, myeloperoxidase; SBPV, systolic blood pressure variability; PP, pulse pressure.

MPO positivity was included as an adjustment variable because elevated MPO levels have been described to be associated with increased all-cause mortality, mainly driven by CV, infectious, and cancer-related causes [26]. To assess the robustness of the association between SBPV and the primary end point, we performed analyses modeling SBPV as a continuous variable. In univariate Cox regression, SBPV was significantly associated with the primary end point (Table 4, HR 8.19, 95% CI 2.07–32.29; *P* = .01). A Cox regression comparing patients in the highest SBPV tertile with those in the combined lower two tertiles is reported in Table S3. Higher SBPV remained independently associated with all-cause mortality after adjustment for PP, age, sex, eGFR, proteinuria, and MPO positivity.

## DISCUSSION

The main finding of the work presented here is the identification of SBPV as a strong and independent predictor of all-cause mortality in a multicenter cohort of AAV patients during the maintenance phase of treatment. This relationship remains highly significant even after adjusting for potential risk factors such as age, gender, renal function, MPO positivity, and BP load during follow-up. Regarding mortality outcomes, 14.7% of patients in our cohort died over a median follow-up period of 58 months. Although comorbidities and disease severity at presentation are well-established determinants of early mortality in AAV [25], patients who died during follow-up showed clinical characteristics at diagnosis comparable to those of survivors, including a similar degree of renal impairment (Table 3). The key features associated with mortality, in addition to older age at onset, were higher PP and greater SBPV. Both these BP parameters are indicative of heightened vascular stiffness and, in this context, may result from increased exposure to vascular damage, possibly due to the vascular inflammation itself. Notably, univariate analysis revealed a clear dose–response relationship between SBPV and mortality risk (Table 4), with progressively higher levels of VVV associated with a stepwise increase in adverse events. Nevertheless, we found no linear correlation between mortality and the type of expressed autoimmunity, nor with parameters of inflammation severity or systemic compromise (Table S2).

A plausible explanation for these findings lies in the study design, which focused on patients in the maintenance phase of AAV. Indeed, during the early stages of the disease, acute clinical events are predominantly immune-mediated, as demonstrated by large cohort studies showing that early mortality is largely driven by disease activity and organ involvement [26]. In contrast, in the long term, these disease-related factors progressively lose prognostic relevance, while competing risks, such as CVD, infections, and malignancies, become dominant determinants of survival [27, 28].

Consistently, univariate predictors of death were baseline PP, TAPP, a reduction in DBP, and SBPV (Table S2), all of which are established indicators of impaired vascular compliance [29, 30]. These characteristics help identify patients with a more unfavourable outcome, possibly due to increased vascular stiffness, which makes them less capable of responding to hemodynamic stress related to volume fluctuations and immunosuppressive therapies, even in maintenance regimens.

Beyond age and traditional comorbidities such as diabetes, hypertension, CKD, and obstructive sleep apnea [31, 32], increased BP variability has been linked to several potentially modifiable factors, including smoking, physical inactivity, poor adherence to antihypertensive therapy, and exposure to drugs known to damage the vascular endothelium, such as glucocorticoids, calcineurin inhibitors, and chemotherapeutic agents [33, 34].

Taking this into mind, it is possible that vasculitis itself acts as a pathogenic factor contributing to vascular injury. Indeed, accelerated atherosclerosis has been described in patients with chronic rheumatologic diseases, including AAV, secondary to the immuno-inflammatory mechanisms characteristic of these conditions [35–38]. In light of this interpretation, it could be plausible that patients presenting with higher VVV might have experienced a biologically more severe form of vasculitis. In this regard, the observation that patients with elevated SBPV at the induction were less frequently treated with rituximab rather than cyclophosphamide may represent an indirect indication of more aggressive disease. Moreover, it could be that patients treated with rituximab may have benefited from a steroid-sparing regimen that protected them from vascular damage capable of inducing stiffness and increased SBPV.

At present, however, the absence of validated biomarkers of endothelial or vascular injury in AAV limits definitive mechanistic interpretation, and these hypotheses warrant further investigation.

It should be considered that AAV are uncommon diseases, and this limits the ability to perform large observational studies to assess the impact of traditional and disease-specific risk factors on CVD burden, as well as clinical trials on the long-term CV effects of preventive treatments. Nevertheless, preliminary data on the impact of classical CV risk factors underscore the importance of enhancing CV risk prediction and management during the maintenance phase in real-world settings [34]. Despite the increasing focus on traditional risk factors in AAV [35], the effect of VVV on mortality remains unexplored.

Notably, SBPV appears to capture prognostic information beyond that conveyed by BP load alone. In our cohort, each 5 mmHg increase in PP was associated with an ∼20% higher risk of death, whereas a 5 mmHg increase in SBPV was associated with a two-fold increase in mortality risk. Moreover, SBPV remained independently associated with all-cause mortality after adjustment for age, sex, renal function, proteinuria, MPO positivity, and PP (Table 4 and Table S3), indicating that SBPV provides prognostic information beyond conventional BP measures. Moreover, the trajectory of renal function during both the SBPV assessment period and follow-up was comparable across SBPV groups, suggesting that the observed association between SBPV and mortality was not driven by differences in renal function decline.

Importantly, rather than proposing a rigid threshold, our findings indicate that SBPV should be interpreted as a continuous risk marker, whereby progressively higher levels of variability confer incremental risk [39]. In this context, the observation that an SBPV >13 mmHg—corresponding to the lower boundary of the highest SBPV tertile—is associated with a significant increased risk of mortality in patients with AAV underscores the clinical relevance of frequent BP monitoring and careful assessment of its variability.

SBPV, in addition to identifying patients with increased vascular stiffness—who may benefit most from smoking cessation—should also prompt intensification of antihypertensive therapy, particularly through the use of long-acting medications such as calcium channel blockers [40]. Furthermore, our results underscore the need to minimize the use of steroid therapies and immunosuppressants or chemotherapies that are prone to causing endothelial damage [41].

This study has limitations inherent to its retrospective observational design, precluding causal inference. The relatively small sample size and limited number of events may increase the risk of overfitting in multi-variable models; therefore, these analyses should be interpreted with caution and considered exploratory. Accordingly, our findings should be considered hypothesis-generating and require confirmation in larger prospective cohorts. Additional limitations include the lack of data on disease activity scores, platelet count, immunosuppressive regimen and dosing, longitudinal changes in renal function, and incident CV events, including cause-specific and CV mortality. Data on ambulatory blood pressure monitoring, cumulative glucocorticoid exposure, and longitudinal changes in antihypertensive therapy were not available, limiting a more comprehensive assessment of factors potentially influencing BP variability. Conversely, the multicenter design, rigorous BP variability assessment, exclusion of the induction phase, prolonged follow-up, and requirement for multiple BP measurements strengthen the validity and clinical relevance of our findings in a real-life AAV maintenance cohort.

In conclusion, our work provides the first consistent evidence that increased VVV confers a significantly greater risk of death in AAV patients during maintenance therapy. Despite advances in immunosuppressive treatment, the persistently elevated CV risk in AAV continues to adversely affect survival. In the absence of reliable tools to identify residual CV risk, BP variability emerges as a promising and clinically accessible prognostic marker. From a practical standpoint, these findings support the integration of BP variability assessment into long-term management strategies aimed at stabilizing BP and mitigating CV burden in AAV.

## Supplementary Material

sfag356_Supplemental_File

## Data Availability

Data obtained through this study and presented in this manuscript may be provided on request. Data shared will be coded, with no personal health information included. The data dictionary and study protocol (with statistical analysis plan) will also be available.
