# Supplementary material for: Blood pressure variability associates with mortality risk of patients with ANCA-associated vasculitis
Source: Clin Kidney J. 2026 Oct 7;19(10):sfag356. doi: 10.1093/ckj/sfag356 (PMC13678739; doi:10.1093/ckj/sfag356)
Supplement: Supplementary file 1 [file sfag356_supplemental_file.pdf]

SUPPLEMENTARY MATERIAL

Supplementary Table 1. Patient Characteristics at AAV diagnosis by mortality status.

Variable	All	Deceased	Survivors	P
**N**	**95**	**14**	**81**	
**Female gender, n (%)**	47 (49)	8 (57)	39 (47)	0.57
**Age at onset, years**	**60.75±16.14**	**68.64±13.61**	**59.37±16.22**	**0.04**
**Pulmonary involvement**	41 (43)	6 (43)	35 (43)	1.00
**MPO positive**	57 (60)	10 (71)	47 (58)	0.55
**PR3 positive**	30 (31)	4 (28)	26 (32)	1.00
**RRT at onset**	12 (12)	1 (7)	11 (14)	0.68
**Time from diagnosis to first-time SBPV assessment visit, months (IQR)**	11 (9-13)	10 (7-13)	9 (11-13)	0.14
**With hypertension, n (%)**	52 (55)	9(64)	43(53)	0.57
**With diabetes, n(%)**	6 (6)	0 (0)	6 (7)	0.59
**With previous cardiovascular disease, n (%)**	3 (3)	0(0)	3 (4)	1.00
**Creatinine (mg/dL)**	3.84±2.51	3.92±2.61	3.52±1.90	0.83
**eGFR, ml/min/1.73m^2 (IQR)**	16 (22)	14.5(21)	16 (21.6)	0.87
**Proteinuria, g/24h, (IQR)**	1 (1.30)	1.1 (1.1)	0.91(1.3)	0.74
**CPR, mg/L (IQR)**	19.79 (39.7)	21.1(84)	18 (35)	0.78
**C3, mg/dL**	113.41±33.49	113.79±29.31	113.35±34.34	0.97
**C4, mg/dL**	30.69±10.72	33.26±12.12	30.27±10.52	0.41
**Induction therapy**				
**IV CYC**	36 (37)	4 (28)	32 (39)	0.55
**OS CYC**	32 (33)	7 (50)	25 (30)	0.22
**RTX**	41 (43)	5 (35)	36 (44)	0.57
**PEX**	20 (21)	1 (7)	19 (23)	0.28
**Maintenance Therapy**				
**On azatioprine, n(%)**	38 (40)	7(50)	31(38)	0.55
**On MMF, n (%)**	27 (28)	4 (28)	23 (28)	1.00
**On steroid, n(%)**	91 (96)	14(100)	78 (95)	0.28

Abbreviations: CRP: C-reactive protein, eGFR: Estimated glomerular filtration rate (mL/min/1.73 m²), IV CYC: Intravenous cyclophosphamide, OS CYC: Oral cyclophosphamide, RTX: Rituximab, PEX: Plasma exchange, MMF: Mycophenolate mofetil, CRP: C-reactive protein, eGFR: Estimated glomerular filtration rate (mL/min/1.73 m²), SBPV: Systolic blood pressure variability,

Supplementary Table 2. Univariate Cox regression analysis for all-cause mortality.

Variable	HR (CI 95%)	P
**Female sex, yes**	1.25(0.42-3.73)	0.68
**Age at onset, year**	**1.06 (0.01-1.10)**	**0.01**
**Pulmonary involvement, yes**	1.54(0.51-4.63)	0.43
**MPO positive, yes**	1.26(0.39-4.14)	0.56
**PR3 positive, yes**	0.96(0.29-3.14)	0.95
**RRT at onset, yes**	0.87(0.11-6.68)	0.89
**With hypertension, yes**	3.12(0.94-10.31)	0.06
**With diabetes, yes**	0.69(0.09-5.34)	0.72
**Creatinine at onset, mg/dL**	1.01(0.83-1.23)	0.87
**eGFR at onset, ml/min/1.73m^2**	0.97(0.94-1.01)	0.20
**Proteinuria at onset, g/24h**	1.01(0.67-1.51)	0.96
**CPR at onset, mg/L**	1.00(0.99-1.01)	0.48
**C3 at onset, mg/dL**	0.99(0.97-1.01)	0.58
**C4 at onset, mg/dL**	1.03(0.97-1.09)	0.34
**Induction therapy**		
**IV CYC, yes**	0.47(0.14-1.57)	0.25
**OS CYC, yes**	0.95(0.31-2.91)	0.94
**RTX, yes**	1.71(0.53-5.50)	0.36
**PEX, yes**	0.25(0.03-1.97)	0.19
**Maintenance Therapy**		
**On azathioprine, yes**	0.97(0.32-2.90)	0.96
**On MMF, yes**	1.51(0.46-4.95)	0.49
**Time from diagnosis to first-time SBPV assessment visit, month**	0.89(0.84-1.06)	0.19
**Creatinine, mg/dL**	1.27(0.89-1.81)	0.19
**eGFR, ml/min/1.73m^2**	0.97(0.95-1.00)	0.08
**Proteinuria, g/24h**	**1.48(1.00-2.18)**	**0.04**
**Haemoglobin, g/dL**	0.69(0.46-1.03)	0.07
**Uric acid, mg/dL**	0.83(0.48-1.44)	0.52
**Glucose, mg/dL**	1.02(0.99-1.04)	0.12
**Total cholesterol, mg/dL**	0.97(0.92-1.02)	0.32
**SBP, 5 mmHg**	1.07(0.92-1.26)	0.36
**TASBP, 5 mmHg**	1.10 (0.89-1.36)	0.34
**DBP, 5 mmHg**	**0.73 (0.55-0.98)**	**0.04**
**PP, 5 mmHg**	**1.19 (1.02-1.39)**	**0.02**
**TAPP, 5 mmHg**	**1.22 (0.99-1.48)**	**0.05**
**Visit to visit SBP SD, 5 mmHg**	**2.03 (1.29-3.02)**	**0.002**
**Visit to visit SBP SD 1st tertile**	Reference	
**Visit to visit SBP SD 2nd tertile**	3.14 (0.28-34.91)	0.35

**Visit to visit SBP SD 3rd tertile**	**13.78 (1.75-108.21)**	**0.01**
**Number of antihypertensive drugs,**	0.63 (0.26-1.50)	0.30
**On beta-blockers, yes**	1.47 (0.85 -2.30)	0.26
**On RAAS blockers, yes**	0.15 (0.01-1.41)	0.09
**On statin, yes**	0.43 (0.05-3.64)	0.43

Abbreviations: CRP: C-reactive protein, eGFR: Estimated glomerular filtration rate (mL/min/1.73 m²), IV CYC: Intravenous cyclophosphamide, OS CYC: Oral cyclophosphamide, RTX: Rituximab, PEX: Plasma exchange, MMF: Mycophenolate mofetil,, CRP: C-reactive protein, eGFR: Estimated glomerular filtration rate (mL/min/1.73 m²), SBP: Systolic blood pressure, SBPV: Systolic blood pressure variability, DBP: Diastolic blood pressure, PP: Pulse pressure, TASBP: Time-averaged systolic blood pressure, TAPP: Time-averaged pulse pressure, RAAS: renin–angiotensin system

Supplementary Table 3. Univariate and multivariate Cox regression analysis for all-cause mortality.

	UNIVARIATE		MODEL 1		MODEL 2		MODEL 3	
**Variables**	**HR (CI 95%)**	**p**	**HR (CI 95%)**	**p**	**HR (CI 95%)**	**p**	**HR (CI 95%)**	**p**
**SBPV 3rd tertile**	**7.49 (2.05-27.34)**	**0.002**	**6.55 (1.73-24.86)**	**0.006**	**8.99 (2.09-38.72)**	**0.003**	**7.46 (1.68-33.14)**	**0.008**
**PP, mmHg**	**1.19 (1.02-1.39)**	**0.02**	1.02 (0.98-1.06)	0.32	0.99 (0.93-1.04)	0.69	1.00 (0.94-1.07)	0.88
**Female sex**	1.25(0.42-3.73)	0.68			2.54 (0.73-8.82)	0.14	2.41 (0.49-11.77)	0.27
**Age at onset, years**	1.06 (0.01-1.10)	0.01			1.04 (0.97-1.11)	0.99	1.02 (0.95-1.10)	0.51
**eGFR, ml/min/1.73m^2**	0.97(0.95-1.00)	0.08					0.99 (0.95-1.04)	0.86
**Proteinuria, g/24h,**	**1.48(1.00-2.18)**	**0.04**					1.18 (0.86-1.62)	0.30
**MPO positive**	1.26 (0.39-4.14)	0.56					1.58 (0.37-6.74)	0.53

Abbreviations: CI, confidence intervals; eGFR: Estimated glomerular filtration rate (mL/min/1.73 m²), HR, hazard ratio; MPO: myeloperoxidase, SBPV: Systolic blood pressure variability, PP: Pulse pressure
